# JDM update September 2014

**DOI:** 10.1186/1546-0096-12-S1-I18

**Published:** 2014-09-17

**Authors:** Clarissa Pilkington

**Affiliations:** 1 Paediatrics, Great Ormond Street Hospital, London, UK

## Aim

To review work in the field of JDM over the last 10 years, and to give an update on the progress of work that is being undertaken in 2014.

## Introduction

JDM is the commonest inflammatory myopathy of childhood and has a wide variation in disease severity and disease course. Collaborative work has moved forward the understanding and the management of JDM in the last 10-15 years.

## Review of international collaborative work

International groups have developed tools for the assessment of muscles, as well as the assessment of disease activity and disease damage. This has allowed physicians to standardise their patient assessments. Core outcome variables for JDM have been proposed (IMACS and PRINTO) as well as definitions of disease flare and remission. Work is going on to agree definitions of improvement to help future treatment trials.

Diagnostic criteria continue to be based on those of Bohan and Peter, but an international survey in 2006 proposed adding items such as MRI and capillaroscopy. An IMACS collaboration has produced a proposed new Classification Criteria which should allow more accurate delineation of cases for future research.

This international work has enabled the first international RCT in treatment-resistant DM and JDM to be undertaken, and for PRINTO to conduct a trial of treatment in new onset cases of JDM. National collaborative efforts have led to setting up registries and cohort studies, allowing better understanding of variation in disease and variation in disease management.

Consensus work has also produced a muscle biopsy score, treatment guidelines to allow comparison of patients within normal clinical practice (CARRA), whilst the SHARE project will produce a European concensus on treatment advice. Work is being undertaken to produce an MRI scoring system; as well as work to agree the minimum data that needs to be collected by physicians caring for JDM (i.e. minimum standards of care).

Laboratory research on myositis specific autoantibodies in JDM is beginning to define different subgroups of JDM patients. This may lead to a better stratification of disease severity and prognosis. This would enable physicians to tailor their patient’s treatment, hopefully improving long-term outcomes.

## Disclosure of interest

None declared.

